# Pulsatile dry cupping in patients with osteoarthritis of the knee – a randomized controlled exploratory trial

**DOI:** 10.1186/1472-6882-12-184

**Published:** 2012-10-12

**Authors:** Michael Teut, Stefan Kaiser, Miriam Ortiz, Stephanie Roll, Sylvia Binting, Stefan N Willich, Benno Brinkhaus

**Affiliations:** 1 Institute for Social Medicine, Epidemiology and Health Economics, Charité University Medical Center, Luisenstr. 57, 10437, Berlin, Germany

**Keywords:** Cupping, Complementary and alternative medicine, Randomized controlled trial, Knee osteoarthritis

## Abstract

**Introduction:**

Cupping is used in various traditional medicine forms to relieve pain in musculoskeletal diseases. The aim of this study was to investigate the effectiveness of cupping in relieving the symptoms of knee osteoarthritis (OA).

**Methods:**

In a two-group, randomized controlled exploratory pilot study patients with a clinically and radiological confirmed knee OA (Kellgren-Lawrence Grading Scale: 2-4) and a pain intensity > 40 mm on a 100 mm visual analogue scale (VAS) were included. 40 Patients were randomized to either 8 sessions of pulsatile dry cupping within 4 weeks or no intervention (control). Paracetamol was allowed on demand for both groups. Outcomes were the Western Ontario and McMaster Universities Osteoarthritis (WOMAC) score, the pain intensity on a VAS (0 mm = no pain to 100 mm = maximum intensity) and Quality of Life (SF-36) 4 and 12 weeks after randomization. Use of Paracetamol was documented within the 4-week treatment period. Analyses were performed by analysis of covariance adjusting for the baseline value for each outcome.

**Results:**

21 patients were allocated to the cupping group (5 male; mean age 68 ± SD 7.2) and 19 to the control group (8 male; 69 ± 6.8). After 4 weeks the WOMAC global score improved significantly more in the cupping group with a mean of 27.7 (95% confidence interval 22.1; 33.3) compared to 42.2 (36.3; 48.1) in the control group (p = 0.001). After 12 weeks the WOMAC global score were still significantly different in favor for cupping (31.0 (24.9; 37.2) vs. 40.8 (34.4; 47.3) p = 0.032), however the WOMAC subscores for pain and stiffness were not significant anymore. Significantly better outcomes in the cupping group were also observed for pain intensity on VAS and for the SF-36 Physical Component Scale compared to the control group after 4 and 12 weeks. No significant difference was observed for the SF-36 Mental Component Scale and the total number of consumed Paracetamol tablets between both groups (mean 9.1, SD ± 20.0 vs. 11.5 ± 15.9).

**Conclusion:**

In this exploratory study dry cupping with a pulsatile cupping device relieved symptoms of knee OA compared to no intervention. Further studies comparing cupping with active treatments are needed.

**Trial registration:**

Clinicaltrials.gov Identifier: NCT01057043

## Background

Osteoarthritis (OA) is a clinical syndrome of joint pain accompanied by varying degrees of functional limitation and reduced quality of life. It is one of the leading causes of pain and disability worldwide [1]. Pathological changes include localized loss of articular cartilage and new bone formation in places of destructive bone loss at joint margins [1]. The most common location for OA is the knee joint, followed by the feet and hips, and it becomes more common with age and women are more affected than men [2]. The treatment focuses on the reduction of pain and stiffness, and the maintenance or improvement of join function; further aims of treatments are the delay of the progression of joint damage and improving the patient’s quality of life [3]. Pharmacological treatments include anti-inflammatory medications such as nonsteroidal anti-inflammatory drugs, which are frequently used to treat the symptoms and are frequently associated with side effects [2,3]. Because oral medication often does not lead to an adequate clinical response in OA, non-pharmacological therapies such as exercise, weight reduction, and physical therapies play an important role in the long-term management of osteoarthritis and are recommended in guidelines. In addition, complementary and alternative medicine (CAM) treatments such as acupuncture or herbal medicine are used frequently by OA patients.

Cupping is one of the oldest known medical therapies. The first descriptions of cupping in the west date back to the famous Egyptian “Ebers Papyrus” (1550 BC) and it was also used in ancient Greek medicine [4]. In addition, cupping is used in traditional Asian medical systems such as Ayurveda, Chinese, Tibetan and Oriental Medicine. In Europe it was widely taught and used from medieval times by monastery treatment providers up to the 19^th^ century by physicians. Throughout the centuries, it was also commonly used by folk healers and laymen but also by medical doctors. It was not until recently, in the 20^th^ century following the development of modern pharmaceuticals, that cupping more or less vanished from mainstream western medicine. Today it is primarily practiced by naturopaths and other CAM treatment providers. Cupping is used for a wide range of diseases. In ancient Greece, Hippocratic physicians recommended it for the treatment of musculoskeletal diseases of the back and extremities, gynecological complaints, pharyngitis, ear ailments and lung diseases [4]. From the therapeutic-principle perspective cupping is a sucking method [5]. The cupping glass is applied to a predefined skin area of the body and a vacuum is generated by mechanically withdrawing or thermally cooling the trapped air under the cup. The skin is then sucked into the cupping glass, resulting in a reddening and warming of the affected area due to increased perfusion. When the vacuum is strong, signs of sub- and/or intracutaneous bleeding (petechiae) may appear. There are two general forms of cupping: dry and wet cupping. In dry cupping only the vacuum is applied; in wet cupping, the skin under the cups is pricked with needles or a scalpel and the blood is sucked into the cup. Pulsatile cupping is a modernized technology using a mechanical device that generates a pulsatile vacuum with a pump. Traditionally cups made of glass, metals or even bamboo are used for cupping, but they do not allow the complete cupping of a big joint, like the knee. Today flexible silicone cups allow a complete cupping also of big joints.

Although cupping has a long tradition there is only limited evidence of its effectiveness. However, recent clinical studies have reported positive results of cupping in patients with musculoskeletal diseases e.g. lower back pain [6], carpal tunnel syndrome [7], brachialgia paresthetica nocturna [8], cancer pain [9] and chronic neck pain [10-12]. In China Cupping is widely used and two systematic reviews were recently published about the results [13,14].

To date no clinical studies have been published about the effectiveness of cupping in knee OA. Thus, the aim of this exploratory study was to investigate the effectiveness of pulsatile cupping in relieving pain and stiffness and improving quality of life in patients with osteoarthritis of the knee compared to no intervention.

## Methods

### Design

This study was designed as a two-group, parallel, randomized controlled, exploratory, clinical study. All study participants gave their informed consent before inclusion. The study was carried out at two study centers: the Charité Outpatient Department and in an ambulatory surgical clinic. Patients were recruited through newspaper advertisements in Berlin daily newspapers. Study information and pre-screening was undertaken by phone by an experienced study nurse. Patients fulfilling the prescreening criteria were invited for a personal consultation with the study physician for information, informed consent, inclusion or exclusion as a study participant, and baseline assessment. Patients were allocated to treatments groups by simple randomization with a 1:1 ratio via a central telephone randomization process. The random allocation sequence was generated by our statistician using SAS 9.2 software (SAS Institute Inc. Cary, NC, USA). Patients were enrolled by the study physician. After signing informed consent and including the patient in the trial an independent study nurse on the telephone line centrally assigned patients to intervention or control according to the randomization list, allocation was concealed. The study protocol was reviewed and approved by the Ethics Committee of the Charité University Medical Center, Berlin, Germany (EA1/230/09; 11.12.2009). The study was registered at ClinicalTrials.gov (NCT01057043).

### Patients

Patients inclusion criteria were: Male and female patients between 40 and 80 years with osteoarthritis of the knee according to ACR criteria (American College of Rheumatology) [15], X-ray classification: Kellgren-Lawrence Grading Scale: 2 – 4 [16,17], and subjective pain intensity at baseline > 40 mm on the visual analogue scale and no other OA therapy except NSAID in the previous 4 weeks. Patients were excluded if they fulfilled one or more of the following criteria: current use of anticoagulants (e.g., Phenprocoumon, Heparin), coagulopathy, or any form of cupping therapy in the previous 12 months; intra-articular injection of corticosteroids or NSAID into the knee joint in the previous 4 months; arthroscopy of the knee joint in the previous 12 months, use of systemic corticosteroids in the previous 4 weeks; physical therapy, leeches or acupuncture in the previous 4 months or other CAM therapies for osteoarthritis in the previous 4 weeks.

### Study interventions

**Figure 1:**
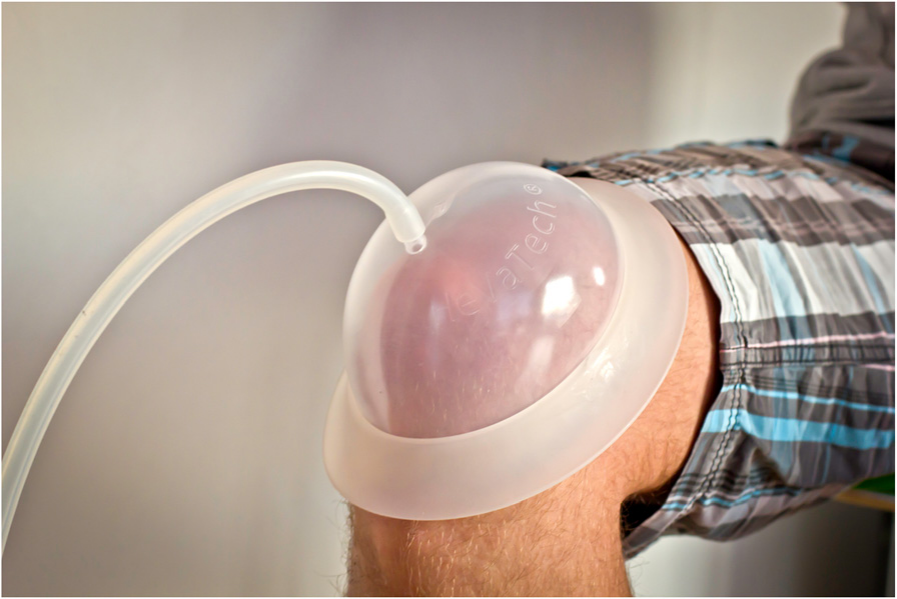
Application of the adaptable silicone cup at the knee.

The intervention protocol was developed *a priori* by a panel of experienced cupping experts. Patients randomized to the intervention group received 8 sessions of pulsatile dry cupping within 4 weeks, twice per week. In addition to cupping the knee, the experts recommended cupping the lumbosacral area in the same treatment session. The traditional cupping experts argued that from their clinical experience this might increase the total effect, but they had no theoretical explanation. They recommended a frequency of 8 sessions in 4 weeks. Pulsatile cupping was administered by a mechanical cupping device (PRV02, HeVaTec® GmbH) with flexible silicone cups to the knee joint and plastic glasses to the skin of the lower back region. The silicone cups allow the cupping of a complete knee joint. The device generates a pulsatile vacuum with a pump. The silicone cups treatment was started with a cupping of the lower back area with 2 cups on each side for 5 minutes, followed by cupping the entire affected knee with a big adaptable silicone cup for 10 minutes (vacuum: 100 – 200 mbar, interval: 2 seconds, pulse: 30-50%) (Figure 1). The cupping device was never tested in a randomized controlled trial before.

Patients randomized to the control group received no cupping intervention for the duration of the study (12 weeks). Patients in both groups were allowed to take Paracetamol on demand with a maximum dosage of 2 g/day according to the NICE guidelines [1] for 4 weeks. All control-group patients were offered a free complete cupping treatment of 8 sessions after the trial was terminated.

### Data collection and outcome parameter

All patients completed standardized questionnaires including all outcomes at baseline, and after 4 and 12 weeks. In cases of bilateral osteoarthritis, the knee defined as more painful at baseline was the one assessed throughout the entire study. The Western Ontario and McMasters Universities Osteoarthritis Index (WOMAC) [18-20] was used to assess pain, stiffness, and physical functioning (higher scores indicate worse symptoms). Pain intensity was measured on a visual analogue scale (VAS) (0 = no pain to 10 = maximum intensity). Generic Quality of Life (QoL) was measured using the SF-36 questionnaire (higher scores indicate higher QoL) [21]. All patients allocated to the intervention group were asked to rate the clinical effect after 12 weeks (improved, not changed, aggravated). As this was an exploratory pilot study, no outcome was defined as the primary outcome. The use of Paracetamol was documented by patients in a diary during the first 4 weeks of the treatment period. Any adverse and serious adverse events were monitored throughout the study by the physicians and the patients.

### Statistical analysis

Due to the exploratory design of the study, no single primary outcome was defined and no formal sample-size calculation was performed. A decision to include 40 patients (about 20 per treatment arm) seemed feasible (organization, funding) in this setting.

The analyses of WOMAC scores, pain (VAS), and SF-36 scales after 4 and 12 weeks were performed by analysis of covariance (ANCOVA) adjusting for the respective baseline value on the intention-to-treat population. Adjusted means with 95% confidence intervals (CI) are presented. Resulting p-values for treatment-group effects are considered explorative. For the use of Paracetamol, descriptive measures (mean, standard deviation (SD)) are given.

All analyses were performed in SAS 9.2 (SAS Institute Inc. Cary, NC, USA).

## Results

**Figure 2:**
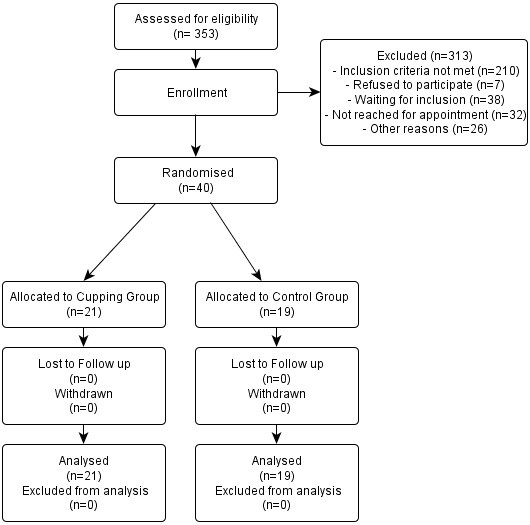
Trial flow chart.

Patients were recruited between January and July 2010. Treatment and follow-ups of the patients were completed by March 2011. Altogether 353 patients were screened for eligibility, 313 of which were not included (main reasons: knee pain due to other diagnoses, severity of osteoarthritis not meeting inclusion criteria) (Figure 2). Of the 40 randomized patients, 21 were allocated to the cupping group and 19 to the control group. The mean age of the patients was 68.1 ± 7.2 in the cupping- and 69.3 ± 6.8 in the control group (Table 1). The pain intensity on the visual analogue scale in both groups was generally high with mean values of about 60 mm in both groups. Only small differences between the groups were found: There were fewer male patients in the cupping group (n = 5, 23.8%) than in the control group (n = 8, 42.1%), the Quality of Life SF 36 mental component scale was higher in the cupping group (58.2 ± 7.2) than in the control group (51.1 ± 11.1) and the WOMAC stiffness subscore was lower in the cupping group (43.1 ± 26.9) than in the control group (50.3 ± 22.3).

**Table 1:** Baseline characteristics of patients in both study groups (values are means and standard deviations except for gender (n, %))

**Characteristics**	**Cupping**	**Control**
	**(n = 21)**	**(n = 19)**
**Age**	68.1 ± 7.2	69.3 ± 6.8
**Gender (Male; n (%))**	5 (23.8)	8 (42.1)
**BMI**	29.0 ± 4.6	28.1 ± 5.5
**VAS Pain**	60.2 ± 12.2	57.9 ± 8.0
**WOMAC**		
- Global Score	39.1 ± 16.2	41.7 ± 15.2
- Pain Subscore	37.4 ± 17.3	40.2 ± 15.3
- Stiffness Subscore	43.1 ± 26.9	50.3 ± 22.3
- Physical Function Subscore	39.1 ± 17.2	41.1 ± 16.6
**SF-36**		
- Physical Component Scale	30.6 ± 8.5	32.2 ± 8.9
- Mental Component Scale	58.2 ± 7.2	51.1 ± 11.1

*BMI* = Body Mass Index, *WOMAC* = Western Ontario and McMasters Universities Osteoarthritis Index (higher scores indicate worse condition), *VAS* = Visual Analogue Scale (0 mm = no pain to 100 mm = maximum intensity), *SF* 36 = The Short Form (36) Health Survey (higher scores indicate higher QoL).

**Figure 3:**
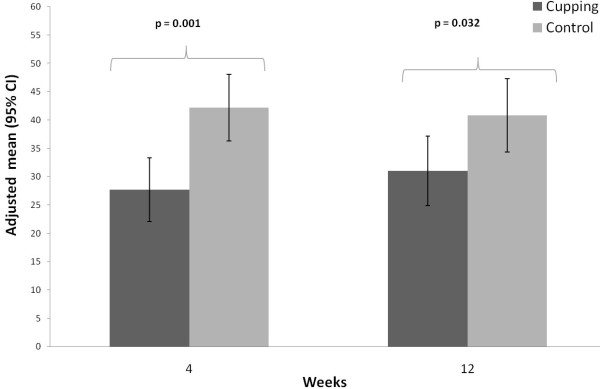
WOMAC global score at 4 and 12 weeks per treatment group (adjusted means and 95% confidence intervals; adjusted for baseline WOMAC global score).

**Figure 4:**
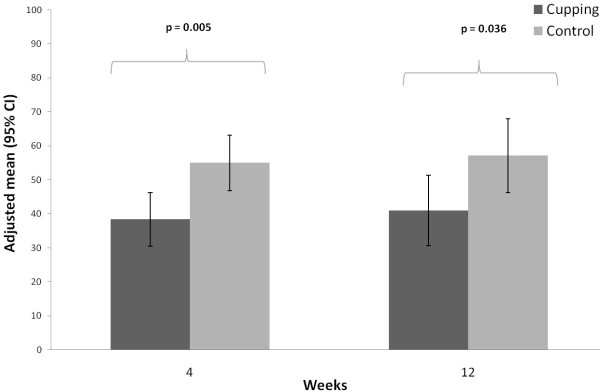
Visual Analogue Scale at 4 and 12 weeks per treatment group (adjusted means and 95% confidence intervals; adjusted for baseline VAS).

After 4 weeks the adjusted mean of the WOMAC global score was 27.7 (95%-confidence interval 22.1; 33.3) in the cupping group which was significant better (p = 0.001) than for the control group, which had a score of 42.2 (36.3; 48.1). After 12 weeks, the WOMAC global score was still significantly better (p = 0.032) in the cupping group (31.0 (24.9; 37.2)) compared to the control group (40.8 (34.4; 47.3)) (Table 2, Figure 3). In addition, the WOMAC subscores for pain, stiffness and physical function showed significantly better outcomes in the cupping group after 4 weeks, but not for pain and stiffness after 12 weeks (Table 2). Significantly lower pain intensity on the VAS was observed in the cupping group after 4 weeks (38.4 (30.5; 46.2)) vs. 55.0 (46.8; 63.2) in the control group (p = 0.005), and after 12 weeks (41.0 (30.7; 51.4) versus 57.2 (46.3; 68.0), respectively, p = 0.036) (Table 2, Figure 4).

**Table 2:** Outcome measures at 4 weeks and 12 weeks (adjusted means and 95% confidence intervals; adjusted for respective baseline value)

**Outcomes**	**Cupping (n = 21)**	**Control (n = 19)**	**p-value**
	**Mean (95% CI)**	**Mean (95% CI)**	
**At 4 weeks**			
**WOMAC Global Score**	27.7 (22.1; 33.3)	42.2 (36.3; 48.1)	0.001
- WOMAC Pain Subscore	25.8 (19.3; 32.3)	40.2 (33.4; 47.1)	0.004
- WOMAC Stiffness Subscore	37.2 (28.6; 45.8)	50.2 (41.2; 59.3)	0.041
- WOMAC Physical Function Subscore	27.0 (21.4; 32.6)	42.1 (36.2; 47.9)	0.001
**VAS Pain**	38.4 (30.5; 46.2)	55.0 (46.8; 63.2)	0.005
**SF 36 - Physical Component Scale**	36.0 (33.5; 38.6)	31.9 (29.2; 34.6)	0.030
**SF 36 - Mental Component Scale**	56.0 (52.3; 59.7)	52.7 (48.8; 56.6)	0.233
**At 12 weeks**			
**WOMAC Global Score**	31.0 (24.9; 37.2)	40.8 (34.4; 47.3)	0.032
- WOMAC Pain Subscore	30.4 (22.5; 38.4)	40.5 (32.1; 48.8)	0.086
- WOMAC Stiffness Subscore	36.3 (28.4; 44.2)	47.8 (38.4; 56.1)	0.052
- WOMAC Physical Function Subscore	30.4 (24.3; 36.6)	40.3 (33.9; 46.8)	0.031
**VAS Pain**	41.0 (30.7; 51.4)	57.2 (46.3; 68.0)	0.036
**SF 36 - Physical Component Scale**	36.3 (32.9; 39.8)	30.2 (26.6; 33.9)	0.019
**SF 36 - Mental Component Scale**	53.2 (49.5; 56.9)	52.6 (48.7; 56.5)	0.831

*WOMAC* = Western Ontario and McMasters Universities Osteoarthritis Index (higher scores indicate worse condition), *VAS* = Visual Analogue Scale (0 mm = no pain to 100 mm = maximum intensity), *SF* 36 = The Short Form (36) Health Survey (Generic Quality of Life, (higher scores indicate higher QoL).

**Figure 5:**
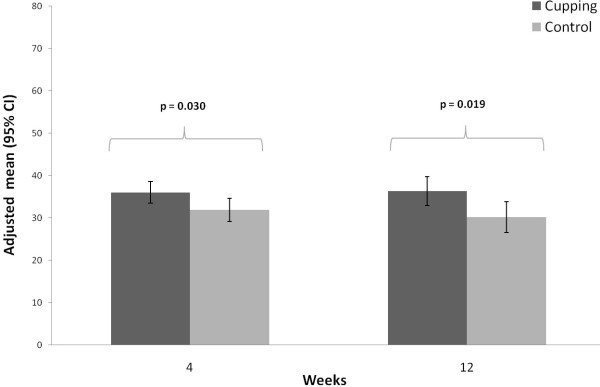
SF 36 Physical Component Scale at 4 and 12 weeks per treatment group (adjusted means and 95% confidence intervals; adjusted for baseline).

**Figure 6:**
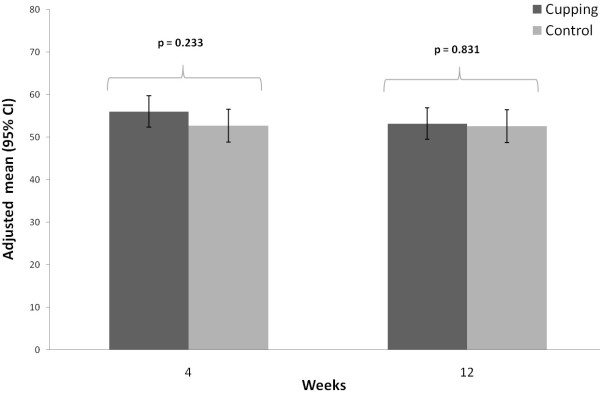
SF 36 Mental Component Scale at 4 and 12 weeks per treatment group (adjusted means and 95% confidence intervals; adjusted for baseline).

The SF-36 Physical Component Scale was significant better (p = 0.030), in the cupping group (36.0 (33.5; 38.6) compared to the control group (31.9 (29.2; 34.6) after 4 weeks, and also after 12 weeks 36.3 (32.9; 39.8) versus 30.2 (26.6; 33.9), respectively (p = 0.019) (Table 2, Figure 5). In contrast no significant group difference was observed for the SF-36 Mental Component Scale after 4 weeks (cupping: 56.0 (52.3; 59.7), control: 52.7 (48.8; 56.6); p = 0.233) and 12 weeks (cupping: 53.2 (49.5; 56.9), control: 52.6 (48.7; 56.5); p = 0.831) (Table 2, Figure 6).

Patients’ pain medication intake was low and comparable in both groups. Patients in the cupping group documented a mean intake of 9.1 (SD ± 20.0) Paracetamol tablets compared to 11.5 (± 15.9) tablets in the control group in the first 4 weeks.

After 12 weeks 10 patients (47.6%) in the cupping group (n = 21) rated the self observed clinical effect as “improved”, 8 patients as “not changed” (38.1%) and 3 patients as “aggravated” (14.3%).

No serious adverse events were observed during the study. Adverse events in the cupping group were mild hematomas in three patients at the skin location where cupping took place, self-limiting light tingling sensations for a few minutes in the legs after cupping the knee in two patients, and an increase of chronic lower back pain in one patient.

## Discussion

To the best of our knowledge this is the first randomized trial evaluating the effect of pulsatile dry cupping in OA of the knee. We observed statistically significant differences in OA patients who received pulsatile dry cupping for 4 weeks compared to patients with no intervention in most of the outcome measures after 4 weeks. Our results are also of clinical importance: There are various minimal clinically relevant absolute changes in OA patients reported in literature: if we consider 9.1 (96% CI: 10.5; 7.5) on WOMAC as a clinically relevant change [22], then our results showed a clinical relevant differences between both treatment groups after 4 weeks. After 12 weeks (including a no treatment period of 8 weeks in both groups) we still observed significant differences, but less prominent, the WOMAC subscores for pain and stiffness were not significant anymore.

In addition, the results of our trial are of high clinical interest regarding the low risk of side effects of the treatment compared to common OA treatments, for example, intra-articular injections of glucocorticosteroids [1].

Due to the exploratory design of our trial, certain limitations have to be considered: We planned an exploratory trial without defining a primary outcome parameter or a formal sample-size calculation – both would be essential in a confirmatory design. Nevertheless, the results of this trial are robust and most of the outcome parameter were statistically significant after 4 and 12 weeks. Another limitation is that our patients were mainly recruited through newspaper advertisements and might not be representative for all patients with OA of the knee. Also, due to the nature of the intervention, a blinding of patients and study physicians was not possible. Because patients in the control group received cupping after 12 weeks of waiting, the long-term effects of cupping in OA could not be properly assessed. However, our results suggest that 8 sessions of cupping in 4 weeks still has a relevant effect after 12 weeks. Only mild side effects were observed, but from a total of only 21 patients in the cupping group, of course only preliminary conclusions can be drawn.

Only a few trials have already evaluated the effectiveness of cupping in various diseases so far. A trial at the University of Essen studied the effect of dry traditional as well as pulsatile dry cupping in chronic neck pain. Statistically significant and clinically relevant effects of cupping compared to waiting-list control were found after 5 sessions of dry cupping with both methods [10,11], but also an effect of traditional cupping on pain and quality of life already after one cupping session has been observed in this trial [12].

Future studies should aim to assess the optimal frequency and application of cupping. As we combined cupping on the lower back and knee region, we are not sure if the cupping of the knee or the lumbosacral area alone or even sham cupping would be as effective.

To minimize various types of bias, blinded trials are needed. The development of a special cupping sham device would be helpful for distinguishing specific from unspecific or placebo effects and local from non-local effects in future studies. The design of our exploratory study with no intervention control group does not allow any distinction between specific and unspecific effects of cupping. Pulsatile cupping itself is already a complex intervention consisting of cupping, massaging and injuring the local (knee) and distant (lower back) skin areas, but also other unspecific factors contributing to placebo effects such as possible relaxing by hearing the rhythmic pulsations and noises of the cupping device, patient-practitioner interactions, empathy, therapeutic expectations and even suggestive effects of the information sheets may play an important role. Comparing cupping with other pharmacological or nonpharmacological therapies would be helpful to validate our results and to assess clinical importance and safety.

In a review about the neurobiological basis of naturopathic reflex therapies including massage, acupuncture cupping and other therapies, Musial et al. [23] summarized three potential mechanisms of actions as hypothesis: Reflex therapies may firstly influence chronic pain locally by deforming or even injuring the skin which stimulates Aβ fibres in painful but also distal skin regions. Secondly, the level of the spinal cord may also be involved: Manipulations may stimulate inhibitory receptive fields of the multi-receptive dorsal horn neurons. Thirdly, therapeutic effects of the special naturopathic setting which may have a relaxing and social comforting effect on the patient and may imitate a “grooming situation” were discussed. The physiological effects of low-amplitude oscillation sucking of skin and underlying tissues have not yet been systematically investigated, but reproducibility has been demonstrated [5].

From a pragmatic point of view, our results indicate that pulsatile cupping can be of valuable clinical use and help for patients with OA of the knee and may therefore be useful in ambulatory health-care services in addition to other analgesic treatment e.g. pain medication on demand.

## Conclusion

Dry cupping with a pulsatile cupping device relieved symptoms of patients with osteoarthritis of the knee and might be a valuable treatment in ambulatory health-care services in addition to other analgesic treatment e.g. pain medication on demand. Further confirmatory research should include a control group receiving sham cupping to investigate the specific efficacy of cupping.

## Competing interests

The authors declare that they have no competing interests.

## Authors’ contributions

Study concept and design: BB, MT, SK and SR. Data management: SB. Intervention: MO, SK, BB, MT. Statistical analysis: SR, SB, SK. Analysis and interpretation of data: SR, MT, BB, SK, MO, SW. Obtained funding: BB, MT, SW. Drafting of the manuscript: MT, BB and SR. All authors read and approved the final manuscript.

## Funding of the study

This study was partly funded by Hevatech GmbH, Grafenberg, Germany.

## Role of the funding source

The sponsor had no influence on the design and methodology of the study, the data collection, analysis or interpretation, or the preparation of the manuscript.

## Pre-publication history

The pre-publication history for this paper can be accessed here:

http://www.biomedcentral.com/1472-6882/12/184/prepub
